# Quality of care in public sector family planning services in KwaZulu-Natal, South Africa: a qualitative evaluation from community and health care provider perspectives

**DOI:** 10.1186/s12913-021-07247-w

**Published:** 2021-11-18

**Authors:** Yolandie Kriel, Cecilia Milford, Joanna Paula Cordero, Fatima Suleman, Petrus S. Steyn, Jennifer Ann Smit

**Affiliations:** 1 MRU (MatCH Research Unit), Department of Obstetrics and Gynaecology, Faculty of Health Sciences, University of the Witwatersrand, Durban, South Africa; 2 School of Public Health and Nursing, College of Health Sciences, University of KwaZulu-Natal, Durban, South Africa; 3 UNDP-UNFPA-UNICEF-WHO-World Bank Special Programme of Research, Development and Research Training in Human Reproduction (HRP), Department of Sexual and Reproductive Health and Research, World Health Organization, Geneva, Switzerland; 4 Discipline of Pharmaceutical Science, College of Health Sciences, University of KwaZulu-Natal, Westville, Durban, South Africa

**Keywords:** Quality of care, Family planning services, Contraception, South Africa, Qualitative research

## Abstract

**Background:**

Quality of care is a multidimensional concept that forms an integral part of the uptake and use of modern contraceptive methods. Satisfaction with services is a significant factor in the continued use of services. While much is known about quality of care in the general public health care service, little is known about family planning specific quality of care in South Africa. This paper aims to fill the gap in the research by using the Bruce-Jain family planning quality of care framework.

**Methods:**

This formative qualitative study was conducted in South Africa, Zambia, and Kenya to explore the uptake of family planning and contraception. The results presented in this paper are from the South African data. Fourteen focus group discussions, twelve with community members and two with health care providers, were conducted along with eight in-depth interviews with key informants. Thematic content analysis using the Bruce-Jain Quality of Care framework was conducted to analyse this data using NVIVO 10.

**Results:**

Family planning quality of care was defined by participants as the quality of contraceptive methods, attitudes of health care providers, and outcomes of contraceptive use. The data showed that women have limited autonomy in their choice to either use contraception or the method that they might prefer. Important elements that relate to quality of care were identified and described by participants and grouped according to the structural or process components of the framework. Structure-related sub-themes identified included the lack of technically trained providers; integration of services that contributed to long waiting times and mixing of a variety of clients; and poor infrastructure. Sub-themes raised under the process category included poor interpersonal relations; lack of counselling/information exchange, fear; and time constraints. Neither providers nor users discussed follow up mechanisms which is a key aspect to ensure continuity of contraceptive use.

**Conclusion:**

Using a qualitative methodology and applying the Bruce-Jain Quality of Care framework provided key insights into perceptions and challenges about family planning quality of care. Identifying which components are specific to family planning is important for improving contraceptive outcomes. In particular, autonomy in user choice of contraceptive method, integration of services, and the acceptability of overall family planning care was raised as areas of concern.

## Background

Quality of Care (QoC) is a key strategy through which human rights can be ensured in the delivery of health care services and thus plays an important role within the sexual and reproductive health (SRH) paradigm, including family planning (FP) services [1]. QoC is a complex concept consisting of various definitions and components. Generally, it can be defined and understood as the process of interaction between the client and the intended standard of care they receive within health care services [2–4]. QoC is distinguished from the concept of access that mainly focuses on all the elements that ensure obtainability of health care services.

Numerous studies have demonstrated the important role that QoC plays in the uptake and continued use of contraception [2, 5–7]. However, evaluating QoC remains a challenge due to the complexity of the concept, which is determined by its definition and the identification of components [2, 8]. A range of frameworks that have been developed over the years adds to the challenge of uniformly assessing QoC across settings [6, 9–12, 81]. Table 1 provides a brief outline of some of the commonly referenced frameworks, proposed definitions, and components of QoC.

**Table 1:** Quality of Care frameworks and components

Authors	Definition	Components
Donabedian (1988)	- Using medical science and technology to improve health without compromising risk. - QoC consists of technical care and interpersonal process.	- Structure - Process - Outcomes
National Academy of Medicine (formally the Institute of Medicine/IOM) (IOM 2001, Lohr 1990) [82]	*“the degree to which health care services for individuals and populations increase the likelihood of desired health outcomes and are consistent with current professional knowledge”*	- Safety - Effectiveness - Patient-centredness - Timeliness - Efficiency - Equity
World Health Organization (2006) [83]	- Comprehensive whole-health system perspective that focus on outcomes for individuals and communities. The working definition includes six dimensions.	- Effectiveness - Efficiency - Accessibility - Acceptability - Equity - Safety
Hanefeld, Powell-Jackson, and Balabanova (2017)	- Quality is a complex concept. - Includes both the demand and supply side of health care.	- Clinical quality - Perceived quality - Process - Responsiveness - Quality as a social construction
Bruce QoC Framework (1990)	- Consists of six elements outlined in a framework. It is both a subjective and outcomes-based concept. It places an important emphasis on the experience of clients.	- Choice of methods - Information given to users - Technical competence - Interpersonal relations - Continuity mechanisms - Appropriate constellation of services.
Bruce-Jain framework revised (2018).	- Revision of the Bruce/Jain QoC Framework with modification to five of the elements originally included. Maintains a human-rights base, client-centred focus.	- Focus on safety of contraceptive products, trained HCPs, and resources. - Choice of methods - Information given to users, to be replaced by information exchange that includes follow-up, and switching methods, service provider or outlet - Technical competence, to include safety. - Interpersonal relations, to emphasise dignity, respect, privacy, and confidentiality - Continuity mechanisms – covered under the information element - Appropriate constellation of services.
NDoH, South Africa National Core Standards for Health Establishments in South Africa (2011).	- The common definition of QoC is to obtain the best possible results with available resources. To attain the goals of health improvement and responsiveness to the expectations of the population.	- Patient Rights - Patient safety, clinical governance, & care - Clinical support services - Public health - Leadership & corporate governance - Operational management - Facilities & Infrastructure
NDoH, South Africa National Contraception and Fertility Planning Policy and Service Delivery Guidelines (2012) [13]	- Uses the common definition of QoC as set out in the National Core Standards for Health Establishments in South Africa.	- Management systems - Accessible and acceptable services - Rights - Continuity of care - Drug management and equipment - Environment of care and infection control

Donabedian [8] outlined three components to consider in the evaluation of QoC (known as the SPO framework): structure, which refers to the characteristics of the setting, such as equipment and human resources; process that includes the activities involved in the actual provision and receiving of care; and outcomes that result due to the provision of care [4, 8]. These three components are still relevant in the contemporary setting and are reflected in numerous other QoC frameworks, including the Policy on Quality in Health Care for South Africa [14, 15].

Within the field of SRH, the QoC framework developed by Bruce [12] and Jain, Bruce [16], known as the FP QoC Framework, is the most popular and widely used [2, 17]. Influenced by Donabedian’s [8] QoC framework approach, the FP QoC framework outlines six components: choice of method, technical competence, appropriate constellation of services, information exchange, interpersonal relations, and follow-up mechanisms [12]. In the FP QoC framework all six elements are grouped under the structure, or preparation, component of the SPO framework, which is then followed by the service-delivery process and the outcomes. Bruce [12] emphasised that the framework should be flexible and adaptable to a variety of settings and that additional components of QoC can be identified according to the context of the setting. In an updated review of the FP QoC framework, Jain and Hardee [2] suggest that safety and information exchange should be added as an expanded definition [2].

The evaluation of QoC is subjective and depends on the value judgements of health care providers (HCP) and users. Bruce [12] highlighted the importance of including the user perspective when evaluating QoC, as this perspective provides key insights into the quality of services that clients receive and their perception of those services. Penchansky and Thomas [18] refer to this as the acceptability of care or services and define it as the relationship between client and HCP attitudes towards each other, and how these attitudes influence the care sought and provided.

In a systematic review of FP QoC in Africa, it was found that the quality of FP stock was the most commonly reported structural factor, while waiting times, provider competence, provision of injectable contraceptive methods, and maintaining privacy and confidentiality were process related factors [19]. Other factors reported under QoC included cost, pre-access criteria, provider workload, and behaviour towards clients (ibid). This systematic review revealed a number of issues in the evaluation of QoC. Firstly, there was significant overlap between factors that related to accessing FP services and components of QoC being evaluated. This could be due to using satisfaction as the outcome measure, therefore providing a skewed evaluation of QoC. Secondly the review highlighted the significant lack of health care provider and community perspectives on QoC.

Due to the complexity of evaluating QoC, satisfaction is often used as the outcome indicator of measure [4, 16, 19, 20], and has been found to be a key determinant for the uptake and continued use of contraception [21]. Satisfaction can be evaluated on the facility, provider, and user levels. It is most commonly assessed through questionnaires or survey data collection methods, although a variety of other techniques have been suggested such as situational analysis, the use of simulated clients and observations [22]. The reliability and validity of satisfaction questionnaires also remain a concern [23], as these methods can be influenced by courtesy and recall bias and result in positively skewed reports about the QoC received and provided [22, 24]. Tumlinson, Speizer [22] showed that there were significant differences in reporting on FP QoC between simulated clients and exit interviews. Although numerous explanations can be raised to explain these differences, it highlights the challenge of reliability of survey or questionnaire data when it comes to QoC evaluation.

Findings from a study in Senegal showed that increased counselling had a negative effect on reported client satisfaction [25]. This was explained by possible poor interactions between the clients and providers – highlighting the importance of the interpersonal relations component. In an earlier review [5] it was also reported that improving the interpersonal relations between clients and providers had the greatest impact on improving FP QoC. A cross-sectional analysis of FP services satisfaction in Mexico found that obtaining the user perspective was crucial to an accurate understanding of what satisfaction with care means [20]. This study showed that interpersonal factors that occur at the point of receiving care (as opposed to access issues which occur prior to received care) had the biggest influence on satisfaction. These factors include adequate information, “feeling that the motive for their visit was addressed”, and “feeling that their opinions had been taken into consideration” [20].

Despite the importance of QoC in FP uptake and use, and the extensive research done in other parts of sub-Saharan Africa, very little data exist on FP QoC in South Africa. To date no other studies have examined FP QoC using the FP QoC Framework, nor explored its definition according to health care providers or community members. This is concerning since South Africa faces numerous SRH problems including high rates of unintended and adolescent pregnancies; high levels of unmet need for FP, and high maternal mortality and pregnancy termination rates [26–28]. In addition, considering the historical political turmoil of South Africa and inequality in accessing quality health care, including contraception, it is important to know whether the restructuring of the health care system has improved the quality of care delivered for all South Africans. This is especially important for FP services since the FP programme has undergone significant restructuring from a specialised vertical programme to a primary health care (PHC) integrated service [29].

What is known about QoC in South Africa is derived from large household and national surveys. Hasumi and Jacobsen [30] reported that over 85% of South Africans were satisfied with their general health care service, but this depended on the type services provided. Private health care clients were significantly more satisfied with care compared to public health care clients [31]. Whether these levels of satisfaction translate into FP services remains unclear. Another study that examined satisfaction with the quality of nursing care found a similar trend of varied reported levels of satisfaction [32]. Findings from other African countries report a similar trend of higher satisfaction with paid-for private health care services than freely provided public services [21].

Numerous reasons have been cited for poor QoC delivered in the public health care sector. Historical political influence; high levels of inequality and poverty; insufficient funding; chronic staff shortages with high staff turnover; and an overburdened service with high demand due to the large burden of disease (HIV and TB epidemics) are some of the major factors identified to contribute to the deterioration of the public health care sector and its ability to deliver quality health care in South Africa [30, 33]. Key policies have been implemented to deal with sub-standard QoC, including the Policy on Providing Quality Health Care in SA and the National Core Standards for Health. While these policies are a key step to improving the care delivered in the public health care sector they tend to focus on the structural issues of QoC and require evaluation to determine their effectiveness and applicability [15, 34].

The integration of FP services into PHC is an important health care strategy for the international community as well as South Africa, especially as a measure to extend access to FP services and Human Immunodeficiency Virus (HIV) care [35, 36]. Despite the importance of integration, little data exist on the influence that integration has on FP QoC. Close, Barden-O’Fallon [37] reported on the effect of integration on FP QoC in Malawi and Tanzania and found that quality improved on the facility, or structure level, but not on the process level. Very few studies have examined the effect of integration on FP QoC in South Africa. This is possibly because the full implementation of FP services with PHC services is still lagging and, in some instances, FP service is still provided as a vertical specialised service [29, 38, 39].

There appears to be a dearth of knowledge about FP QoC in South Africa, with no data from qualitative studies or research using the FP QoC Framework. Even less is known about how HCP and community members define and evaluate FP QoC [40, 41]. A qualitative approach using focus group discussions can mitigate against some of the methodological problems identified with quantitative questionnaires or surveys in the evaluation of FP QoC. The aim of this paper is to explore FP QoC using the FP QoC Framework through a qualitative methodology from a health care provider and community perspective.

### Setting: family planning and contraceptive use in South Africa

An estimated 83% of women who need contraception in South Africa access it through the public health care sector [28, 42]. The current contraceptive prevalence rate (CPR) for South Africa is 54% for married and 64% for unmarried women, a figure that has remained largely stagnant over the past decade [28]. Teenage pregnancies and early age of first birth remain key concerns for the country, with 16% of women aged 15–19 years having begun childbearing. The mean age of first pregnancy is 21 years [28]. Unmet need for contraception remains high at 19% for sexually active women and 15% for married/in-union women [28]. Over half (54%) of all pregnancies for all women who have begun child-bearing are unintended, with 34% of those being classified as mistimed and 20% as unwanted [28]. Factors associated with unintended pregnancies include young age at first pregnancy, being unmarried, and having an HIV unknown or positive status [27, 43–45]. Another important measure is the Couples Year Protection Rate (CYPR) which is also used as a proxy measure for the CPR. The current national target for the CYPR is 75%, however the CYPR for the 2018/19 year was 61% [46].

## Methodology and analysis

### Study design

The study reported on in this paper was conducted in the eThekwini District of KwaZulu-Natal (KZN) province, South Africa. The CYPR for KZN has consistently fallen below the national median, with the 2018/19 CYPR being 44.4% - a significant drop from the 2016/2017 CYPR of 66.1% [46]. KZN also has the largest number of people living with HIV in the country, with over two million of the seven and a half million people living with HIV residing in the province [47]. The eThekwini District is the third largest metropolitan district in South Africa, with an estimated population size of three and a half million people. The CYPR for eThekwini is also below that of the national average at 44.4% for 2018/2019. Similar to the KZN province, eThekwini also has the largest number of people living with HIV per district in the country [47].

This study was conducted as part of formative work to inform the development of an intervention that aimed to increase met need for FP/C through community and HCP participation from a human rights perspective, in South Africa, Kenya and Zambia (the UPTAKE Project) - see Cordero, Steyn [48] for an account of the overall study findings. QoC was identified as a key intermediate outcome for addressing unmet need and was purposefully explored during the formative phase. A qualitative methodology was chosen due to the exploratory nature of this project to uncover factors that influence the uptake of contraception, including aspects such as QoC. In this article, we report on qualitative findings on QoC from South Africa. The methodology was also reported in a previous paper which focused on the male partner influence on contraceptive use [49]. The reporting of the qualitative data results followed the COREQ guideline [50].

In-depth interviews (IDIs) and focus group discussions (FGDs) were conducted and purposive snowball sampling was used to recruit participants [49, 51]. In total, 127 participants were recruited from two areas within the eThekwini District. Area 1 was classified as rural, and Area 2 as urban. Both areas are predominantly populated by Black South Africans who speak isiZulu as their home language [52].

Fourteen FGDs were conducted, twelve with community participants, and two with HCPs between 2015 and 2016. Table 2 shows the breakdown of the participants from the FGDs. IDIs were conducted with eight key informants (KIs) who were purposively selected based on their expertise in FP/C services, or community involvement. Four of the KIs were based within the communities from which the community members were recruited. The remaining four KIs held senior or programme level positions and specialised in the field of SRH and FP/C. Table 3 outlines the composition of the KIs.

**Table 2:** FGD participant breakdown

FGDs conducted	No. of participants (n)
1. Females, urban, teenagers (aged 15–19 years)	9
2. Females, rural, teenagers (aged 15–19 years)	10
3. Females, urban, young adults (aged 20–34 years)	8
4. Females, rural, young adults (aged 20–34 years)	10
5. Females, urban, adults (aged 35–49 years)	8
6. Females, rural, adults (aged 35–49 years)	7
7. Males, teenagers (aged 15–19 years)	10
8. Males, young adults (aged 20–34 years)	8
9. Males, adults (aged 35–49 years)	7
10. Females who are unmarried, single (20–34 years)	8
11. Females who are married/in a relationship > 1-year (20–34 years)	10
12. Females with no children (who are not infertile) (18–49 years)	8
**Total community participants**	**103**
13. HCP from local health facilities (including management, professional nurses): Group 1	8
14. HCP from local health facilities (including enrolled nurses, counsellors, and other operational staff): Group 2	8
**Total HCP participants**	**16**

**Table 3:** Key Informant participant breakdown

Key informants	No. of participants (n)
1. Education	1
2. Community Care Givers	2
3. Traditional Healer	1
4. Programme Managers working in sexual and reproductive health (SRHR)	4
**Total key informants**	**8**

FGDs and IDIs were conducted by trained research team members who had experience with qualitative interviewing and who were fluent in local languages (isiZulu and English). Interviewers were appropriately matched with gender, age, and professional position to facilitate discussions. Demographic data were collected from all participants and were descriptively analysed. FGDs were conducted at community-based facilities. Key stakeholders were interviewed at locations convenient for them, that ranged from the research site’s offices to the participant’s home.

Audio recordings of the FGDs and IDIs were transcribed and translated from isiZulu to English (if necessary). The transcripts were reviewed and checked for accuracy and any ambiguity in the translations were discussed and clarified to ensure accuracy of the translations. The results were shared with community members and key stakeholders at the end of the project. Community members and key stakeholders expressed a high degree of agreeability with the shared findings, thus contributing towards the validity and accuracy of the data.

Interview guides contained key theme-specific questions that were tailored for each category type of participant, including: female FGDs, male FGDs, HCP FGDs, and key stakeholder IDIs. Similar key theme questions explored understandings of family planning; knowledge, attitudes, and practices; key barriers and enablers to family planning access; perceptions and definitions of quality of care; and the role of community participation in family planning and contraceptive services.

The study approach is supported by the social constructionist stance that emphasises the equal value of various perspectives on the same topic. Obtaining these various perspectives offers a robust and holistic view of QoC within the provision of family planning and contraceptive services.

### Ethics approval and consent to participate in the study

The development and conduct of this study followed the principles of ethical conduct in research studies involving human participants as outlined by the Declaration of Helsinki. All researchers, research assistants, and interviewers involved in the conduct of this trial had valid Good Clinical Practice training and certification prior to collecting data. This study received World Health Organisation (WHO) Ethics Review Committee (ERC) and Research Project Review Panel (RP2) approval (Project ID A65896). Local country ethics review and approval was provided by the University of the Witwatersrand Human Research Ethics Committee (Health, reference no M1504101). Further ethics reciprocity was provided by the University of KwaZulu-Natal’s Biomedical Research Ethics Committee (BREC). Permission was also obtained from the KwaZulu-Natal Provincial Department of Health to interview HCPs. All participants voluntarily signed an informed consent form, which included permission to audio record the interview sessions. Consent for the minors (those aged < 18 years) to participate in the study was obtained from their identified parents or guardians. Assent to participate was obtained from the minors themselves.

### Analysis

A master code book was developed amongst the researchers in all three countries in which the larger study was conducted [51]. This team approach allowed for rich discussion about the meaning of concepts and codes, further establishing the validity and appropriateness of the code list. Independent coders double coded a subset of the data to increase reliability of the data [51]. NVivo (version 10, QSR International) was used as the computer assisted qualitative data analysis software that facilitated coding and analysis of the data.

Thematic content analysis of the qualitative data was done using a social constructionist approach. The social constructionist approach argues that the lived experience that contains actions and consequences constitutes what is real and truthful for people [53, 54]. This theoretical lens was useful in uncovering individual perceptions and interpretations about the meaning of QoC.

A priori themes along with inductive emergent themes were elucidated from the larger data set surrounding the topic of FP QoC. For the inductive part of the coding analysis, the constant comparison method was used to explore the data and develop the initial coding into categories and themes once data saturation was reached [55]. A priori themes were identified using the Bruce-Jain FP QoC framework and Penchansky and Thomas’s definition of access to healthcare [12, 16, 18]. According to the FP QoC framework, the evaluation of FP QoC can be divided into structure and process. The structure component can consist of choice of method, technical competence of the providers, and appropriate constellation of services [2, 8]. The process component comprises of the provision of information, interpersonal relations, and follow-up mechanisms [2, 8]. An in-depth discussion on the overall methodology used in this project is described elsewhere [51].

## Results

### Demographic results – community male and female participants

A total of 103 community participants participated in this study. Seventy-eight of the community participants were female, and 25 were male. The mean age of female community participants was 26.4 years, and males 23.8 years. The majority of female participants (*n* = 48, 62%) reported having a regular partner but not living together, compared to 28% (*n* = 7) of male participants reporting the same. Only female community participants reported being married (*n* = 2, 4%), while 16% (n = 4) of male participants reported having casual partners.

At least one prior pregnancy was reported by 66.6% (*n* = 52) of female participants of which 86.5% (*n* = 45) were unintended and only 15.3% (*n* = 8) were planned. Thirty-two percent (n = 8) of male participants reported at least one pregnancy with a female partner with 87.5% (*n* = 7) of those pregnancies being unintended. Only one male participant reported having a planned pregnancy with a female partner.

Amongst the community participants the majority (*n* = 86, 83.5%) reported modern contraceptive use. The remainder (*n* = 17, 16.5%) reported not ever having used contraception, with the majority of those being adolescents (*n* = 12, 70.5%). Multiple method use (condoms and a hormonal contraception) was reported by 38.4% (*n* = 33) of the participants. None of the participants reported using traditional methods such as thigh sex, lactational amenorrhoea method, withdrawal, or fertility awareness, as contraceptive methods.

### Demographic results - health care providers and key informants

HCP FGD group 1 (management and professional nurses) consisted of six female and two male participants. The mean age of these HCPs was 39.2 years, and the average years of experience in their current position was 4.9 years. In HCP FGD group 2 (enrolled nurses, counsellors, and other operational staff) all participants were female (*n* = 8). The average age was 37.6 years and the average years of experience in their current position was 6.4 years. The Key Informants (Table 3) consisted of seven females and one male, with the mean age being 51.2 years. The average years of experience in their current position was 10 years.

### Thematic results

The thematic results were grouped into two broad thematic categories following the FP QoC framework. These thematic categories included the definition of FP QoC and evaluation of FP QoC. The evaluation category was divided between the structure and process components.

### ﻿Definition of family planning quality of care

In general community participants expressed some confusion about how they defined FP QoC. Most community participants discussed the quality of the methods provided to them, using dual protection, outcomes, and the attitudes of HCPs.

One rural adult female expressed her opinion, citing outcomes:

*P: In my opinion I am not sure of good quality since injection is...since some have said that they got pregnant while on the injection [...] And others said that the condom bursts. It helps, but there is nothing that you can say is good quality in my opinion.*

*[Rural Adult Females, FGD, P1]*

A young adult male explained what he thought constituted quality care:

***I: What type of assistance can you say is good quality services, of care at the clinic?***

*P: ... the most important thing my brother is the way you are treated.*

*[Young Adult Males, FGD, P4]*

HCP had more robust definitions of QoC, focusing more on infrastructure, training, and HCP attitudes:

*P8: I think good quality is whereby you have a room with an allocated sister (nurse) doing family planning whereby each and every client that comes for family planning will just go straight and get her health education and start injections. That’s how we can say we are having quality.*

*P6: I think I agree with number 8, but that sister (nurse) needs to also have been trained very well with regards to family planning [ … ]. And also, the attitude of that sister would also have to be youth friendly. [HCP Group 1, FGD]*

### Evaluation of FP QoC: structural and process themes

FP QoC was assessed according to the structural and process related a priori themes as outlined in the FP QoC framework. While these a priori themes provided guidance, emerging sub-themes were also raised and grouped into each category.

#### Structural category – evaluation of health facility readiness

Sub-themes that emerged under the structure category include limited decision-making power, lack of FP trained HCPs, infrastructure, integration of services, and mixing of clients.

##### Limited decision-making power by clients

Community and health KI participants reported that FP clients had limited autonomy to decide which contraceptive method to use as HCPs would choose for them.

One community participant described this situation when she recounted her experience when she initiated contraception:

***F: She [HCP] chose for you?***

*P3: Yes.*

***F: You didn’t choose for yourself?***

*P3: [No] because she said, ‘oh it’s your first time’, I said yes, it’s my first, she said ‘because you are a person that is starting, it’s 3 months’, that’s what she said. She didn’t give me like a certain pamphlet that says, ‘these are the family planning that are available which one do you prefer?’.*

*[Females without children, FGD, P3]*

A health KI also recounted her experience with pre-prepared Intra Uterine Devices (IUDs):

*P: Like I told you, after they [clients] have given birth [ … ] the sisters [nurses] actually come lining up with the injectables. When we actually started to resuscitate the IUD in South Africa I went into a ward once and the sister actually had it drawn up, she had everything prepared -And you know, you can’t prepare [an] IUD ahead of time, so it was all prepared waiting. So, it just tells you that there is no choice.*

*[KI HCP, IDI, P8]*

##### Lack of sufficiently trained HCPs

A key concern for providing QoC was the lack of HCPs specifically trained in the provision of contraception and SRH counselling. HCP KI’s reported that although HCPs received various training, especially in HIV care, they were rarely trained on family planning. One health KI explained:

*P: [I] f you look at how much training is happening for NIMART [Nurse Initiated Management of Anti-Retroviral Therapy] [ … ] for HIV, there is all kinds of training. But what training is happening for family planning or sexual and reproductive health? [ … ] They go on NIMART training, they go on this training, on that, they go on all kinds of training. What has happened to family planning? It all fell away!*

*[KI HCP, IDI, P7]*

One HCP participant explained her working day as the only FP trained nurse in the clinic:

***F: [H] ow many were you [HCPs] when you saw 70 [clients]?***

*P1: I was alone yesterday.*

***P2: [Staff] shortages?***

*P1: So, if we are two, we will see 35, 35 depending on if the person has been trained very well because I am the only one trained and that’s standard in family planning.*

*[HCP Group 1, FGD]*

##### Infrastructure and privacy

HCP participants raised how inadequate infrastructure limited their ability to provide quality care, especially because FP clients require privacy:

***F: [L] ook at your family planning service and then let me know if your facility is offering quality service to your community.***

*P: Hey, infrastructure. We don’t have a separate room for family planning.*

*[HCP Group 1, FGD, P5]*

Another HCP participant raised the issue of privacy:

*P: And those rooms, but they don’t have their room, because a person needs her privacy.*

*[KI Community Member, IDI, P2]*

##### Constellation of services – integrated care

Integration of FP services with PHC services was a much-discussed topic by community and HCP participants. Predominantly participants reported that integration has had a negative effect on the user experience in the PHC when seeking FP services.

According to their evaluation the integration of services has had a negative effect on FP QoC, as one health KI explains:

***I: So how do you think that decentralisation of services has affected the community’s access to family planning?***

*P: This was a vertical programme and it had to be amalgamated, decentralised. That actually killed family planning, as far as I’m concerned.*

*[KI HCP, IDI, P5]*

Another health KI pointed out that the addition of HIV care has overshadowed FP services:

*P: [O] ne person has to do so many programmes [ … ] the [HIV] programme came and overshadowed family planning. I heard that family planning used to be a good, very huge programme here in South Africa, and they were doing well. But with HIV it was overshadowed [ … ]*

*[KI HCP, IDI, P8]*

The integration of services should expand FP services access to a variety of clients, including adolescents. One health KI reported that there is a lack of youth services:

*P: There’s an absolute lack of youth friendly services [ … ] And the staff should be specially trained to manage adolescents.*

*[KI HCP, IDI, P7]*

##### Mixing a variety of clients

A consequence of integrated services is that FP clients are mixed with clients who need different types of care.

*P: Another thing that makes us to be reluctant to go to the clinic is that we are mixed with the sick [clients].*

*[Females without children, FGD, P2]*

HCP participants also picked up on this issue and noted that FP clients had to endure longer queues:

*P: The first thing that poses a difficulty for our clients, it’s when they are well and they don’t want anything else but family planning, then you find that because of integration of services they have to go to a clinic where they have to follow the [same] queue, especially on their first visit, when nobody would know whether they [are] coming for family planning or any other thing.*

*[KI HCP, IDI, P5]*

#### Process related category – evaluation of the process of receiving and giving care

Themes that were raised under the process category included attitudes of providers, fear, lack of counselling/information exchange, and time constraints. Follow-up mechanisms were not raised in the data.

##### Attitudes of health care providers

Interpersonal relations which include attitudes of HCPs towards clients were raised as a key concern in providing quality care.

One HCP participant reflected on this issue:

***I: [H] ow could your facility provide better quality [of care]?***

*P: I think by improving staff attitude, it’s still the problem [ … ]*

*[HCP Group 1, FGD, P1]*

A female community participant explained that poor HCP attitudes were linked to insufficient information and counselling being provided:

*P: VERY POOR! (exactly, says someone) when it comes to family planning. Because now they don’t give you [information]. Like me as I am of this age maybe, I will get there and say I have never been on family planning, they will say: ‘hey you are old!’ So, it’s an attitude [problem and] it just kills your spirit. So, they don’t have patience to give you full information.*

*[Married Females, FGD, P2]*

##### Fear

Related to reports of negative HCPs attitudes were reports of clients feeling fearful of HCPs, and therefore reluctant to engage with FP services.

One community participant from the married/in-union group raised a number of issues, including being afraid of HCPs:

*Yes, we do face problems [ … ]. First [the] clinic is far, secondly the nurses are rude, thirdly this clinic that is here it is very poor, you would just [go] there and they would say there is no injection. And [then you] go [to another PHC] and the nurses there are rude [ … ]. You end up being afraid. How do you go to a place where you [are] asked, why are you on the injection instead [of] behaving yourself, why can’t you close your thighs because you [are] so young?*

*[Unmarried female group, P004]*

Another female community participant from the group without children also raised the issue of fear and being shouted at by HCPs:

*P: Hey, it’s just that the nurses talk, they say bad things and you end up saying you will never go to the clinic again [ … ] you get shouted at when you come to collect this thing [contraception], you are shouted when you are pregnant, which is better then?*

*[Females without Children, FGD, P6]*

##### Insufficient counselling and information exchange

Insufficient provision of FP related counselling and information were raised by numerous community and HCP participants. HCP participants noted that they often did not have adequate information and educational resources to provide adequate information and counselling services to clients. A key informant explained:

***I: What about to access of information, availability of information about the methods?***

*P: Well, that’s another area that we are not very strong on, you know you go to a clinic and there we don’t have a lot of- IEC material that you could readily go.*

*[KI HCP, IDI, P8]*

One female community participant discussed how the quality of services varied between PHC facilities. One important aspect that she mentioned is that FP counselling seemed to fall away once clients are initiated on contraception:

*P: Clinics too are not the same in our communities. Sometimes you go to the clinic and find good service, they have time for their patients, and they educate them about everything. You find that one got educated first time she started injection. When you are initiated, they explain then maybe things she learnt on that day were too many and [she] forgot some. When she comes back for repeat dose nobody has time to educate you, they just inject you and you go home, and you have forgotten other things. Important thing she can do is to remind you to do 1, 2 and 3. So if it can happen maybe there must be other ways of getting more information each time you go to the clinic.*

*[Married Females, FGD, P8]*

##### Time constraints

Time constraints were linked to integrated services, staff shortages, overburdened facilities and impacted crucial care aspects such as providing adequate counselling. One HCP participant outlined how multi-tasking by HCPs resulted in reduced time available to spend per client:

*P: [W] hen you come maybe at 06:00am or 07:00am for family planning they won’t start with you, they will start with other people and then later maybe they consider you and family planning. Then together with staff shortages [it is] a problem because usually the person who is doing the family planning who’s giving the injections is the one who is doing injections for any other prescriptions, and she is [also] the one doing the dressing for anyone who came for dressings. She will do family planning, she will do injections, and she will do the dressings. Yes, it will take time for a [client] to be take [n] care of.*

*[HCP Group 1, FGD, P8]*

Another HCP pointed out that FP care requires additional time due to the counselling involved:

*P: [O] ur clients, they wait for a long time to be seen because they need to be taught about the family planning then from there, they come back to the queue. So, it’s not good quality for the clients [ … ].*

*[HCP Group 1, FGD, P5]*

## Discussion

The results presented in this study provide an important update on the state of QoC delivered in FP and SRH services in South Africa. The main findings of this study showed that key structural and process challenges exist that limit or prevent the delivery of quality FP care. Identifying these challenges are crucial to improve outcomes for contraceptive use. In the original FP QoC framework, all six elements of QoC are grouped under the structure component, however our data showed that it was more beneficial to group the themes according to the structure or process components. This allowed us to highlight how some elements of QoC can influence others and explore what happens during the process of giving and receiving care in this setting. Additional elements were also identified by community members and HCPs that were considered important in the provision of quality FP care. Structural components were shown to influence process components, which highlights the importance of including the user perspective in the evaluation of QoC, and to identify which elements fall under which component [12].

A significant finding was the limited autonomy that FP users have in either choosing to use contraception or which method they prefer. User choice is one of the most important components of QoC because it highlights the human rights aspect in the provision of SRH [1, 2, 12]. In failing to ensure that user choice is protected during the delivery of contraceptive care, basic human rights that are provisioned for in the Constitution and National Health Act 61 of 2003 are not being met [56, 57].

There has been much concern over the limited range of contraceptive methods available in South Africa and the over-reliance on the shorter acting contraceptives such as the DMPA injectable [46, 58]. This concern was addressed by policy updates which encouraged a shift from the shorter acting contraceptives to long-acting reversible contraceptives (LARCS) through the introduction of the Implanon NXT sub-dermal Implant and the revival of the IUD [58, 59]. While more types of contraceptive methods increase choice there is also the risk that some methods will be promoted over others [58]. Individual choice, whether to use contraception and which method to use, forms a crucial part of human rights in SRH [1] and was identified by Bruce [12] as a central component of FP QoC. Both community and HCP participants in this study discussed the limited autonomous choice that clients have when deciding which contraceptive method to use. The limited ability for users to choose their method of preference was linked to a lack of counselling and information. This is not an isolated finding, since only 44% of South African women who use contraception make a fully informed decision [28]. A fully informed decision requires that the user is informed about the variety of methods available to them, what side-effects to expect, and what actions to take in the event that side effects do occur [28].

The overall evaluation of FP QoC was that sub-standard care was being provided to clients who seek FP services from public health facilities. But as discussed in the literature, evaluation depends on how QoC is defined, and which components are deemed important to both community members and HCPs [4, 8]. Community participants were overall unclear on how to define QoC and mainly related it to the outcomes of using contraception. This was evident in reports where participants discussed negative outcomes such as ‘burst condoms’ or falling pregnant while using a hormonal contraceptive. Various factors may also influence outcomes, such as poor user technique, side effects, or poor administration by HCPs who may not be adequately trained in the provision of contraceptive care, as was demonstrated by the introduction of the Implant [60, 61]. HCPs have more robust definitions of what QoC should entail, possibly because they have been trained or familiarised with the concept. HCPs pointed out that infrastructure, provider attitudes, and adequate provision of counselling and information forms important aspects of QoC.

### Structure related components

The results were categorised under the structure or process components of the QoC framework [8, 12]. Under the structure category, numerous sub-themes were raised including a priori sub-themes such as choice of method, competence of HCPs, and appropriate constellation of services. Emergent sub-themes that can be grouped under the structure category because they deal with the readiness of a service to provide quality care, include competent and trained HCPs, infrastructure, time constraints, and mixing of a variety of clients.

Nurses are the main providers of FP and contraceptive services in the public health sector. The restructuring of the South African national health care system required training adjustments for nurses to be more generalist, PHC providers rather than specialist nurses [62]. This change, according to the key informants of this study resulted in decreased numbers of nurses who are specialised in contraceptive care – which has been overshadowed by the impact of HIV/AIDS on the health care system [29, 63]. There is a need for PHC nurses to be well trained and informed about the available contraceptive methods, their mechanism of action, potential side-effects, and interactions with other treatment regimes especially given the concerns around DMPA, increased risk for HIV acquisition, and method switching [64, 65]. Insufficient training of HCPs was reported as one of the reasons for frequent discontinuation and rumours about side effects of the Implant [60]. Although no community participants reported technically incompetent HCPs in this data, there were numerous reports about the lack of adequate information and counselling on the various contraceptive methods which was attributed to limited HCP training. Health care KIs who had observed the change in nursing education and training over the last two decades were particularly concerned about the lack of FP specific training and it is a key area to focus on improving FP QoC.

Another significant issue raised in the data was the state of infrastructure which has been discussed in the literature [31, 66]. Limited infrastructure was grouped under the structural component since it can inhibit the ability of health care facilities to accommodate a variety of clients, including FP clients and adolescents thereby constraining the degree to which a facility can prepare to deliver services. The WHO stipulates the importance of adequate infrastructure to delivery integrated care in their guideline on integrated care [67]. HCPs reported that they worked in facilities where the infrastructure impeded their ability to provide the standard quality care to clients. Poor infrastructure resulted in a variety of the challenges reported in this study, including a lack of privacy for FP clients, mixing of clients needing a variety of services, and increased waiting times. Furthermore, the well-being of HCPs can also be affected by poor infrastructure, resulting in feelings of frustration which can manifest as negative attitudes and behaviour towards clients – a finding also reported in this study [29].

Integration of services can be discussed under the constellation of services component of the FP QoC framework. The need to provide a range of services at PHC facilities is clear, especially the provision of HIV care with FP care considering the high rate of unplanned pregnancies amongst HIV positive young women [27, 45]. The outcome benefits that integration of services can have depends on the successful implementation of integration across the various levels of health services – from policy to the facility level where front-line care is provided [63]. After almost two decades of restructuring, full integration of services is still not complete in the South African public health care sector [29, 62].

The literature reports that integration tends to affect structural FP QoC components more rather than process components [37]. However, findings from this study described the negative effects that integration can have on the structural aspects of FP QoC, as well as on some of the process related factors. This highlights the importance of identifying which factors of FP QoC fall under, either the structural or process components as they are interrelated. Structural QoC components negatively affected by integration in this study include discussions about decreased technically trained HCPs in preference for more generalist trained HCPs; mixing of ill and well clients; and the perceived displacement of FP programmes by seemingly more important services such as HIV care. These structural components influenced process components and were reflected in reports about decreased time available to spend per client, and a lack of counselling.

Another consequence of integrated services is that there is a mixture of clients who attend PHC facilities [39]. FP services differ from all other services in that provision of FP and contraception does not prevent or treat and illness, but rather aids with the planning and prevention of pregnancy [68, 69]. Both the HCPs and community participants raised this dissatisfaction, where seemingly healthy female clients are expected to interact with a variety of clients seeking various health-related services. This dissatisfaction was particularly prominent in community participant reports that described single row waiting structures with clients that they perceived as being un-well. This finding was also described in earlier assessments of the integration of SRH with PHC services however, little seems to have been done to improve this – possibly due to the vague instructions about how integration of services should be managed at the service provision level [29, 39]. The literature further shows that such mixing of clients has resulted in the loss of FP clients [39].

### Process related components

Sub-themes raised under the process category included a priori *sub-themes –* interpersonal relations and lack of counselling/information exchange, while emergent sub-themes were fear and time constraints. There were no discussions about follow-up mechanisms in the data.

The interpersonal relations sub-theme was raised in discussions about the attitudes of HCPs towards clients, and can also be classified under the acceptability component of the access to health care model, which refers to the attitudes and beliefs held by HCPs and users towards each other and the services that are provided or sought out [8, 12, 18]. Negative attitudes towards FP clients held by HCPs who provide FP care were identified as a key concern that influenced the acceptability of care – a finding that is well supported by the literature [70–72]. The community participants raised the importance for FP clients to be treated with respect, and for HCPs to be supportive to a variety of FP users including adolescents. The attitudes of HCPs were identified as a key reason why FP clients may discontinue their contraception use and was identified by both community and HCPs participants as an important area to improve QoC.

Linked to provider attitudes were reports of fear of HCPs as a consequence of negative attitudes and behaviours of HCPs towards FP clients. Younger community participants in particular raised being afraid as a reason not to engage with FP services. Fear is related to health care stigma where HCPs discriminate against clients, especially if they have pre-conceived beliefs about who should use contraception or not [70, 72]. This scenario was also reported in studies about the introduction and implementation of the Implanon NXT Implant in the FP programme [60].

A key aspect for FP clients to make an informed choice is to receive adequate information and counselling (information exchange) about the variety of contraceptive methods available to them, and for counselling to be continuous [2, 37] . Close, Barden-O’Fallon [37] found that counselling substantially decreased with subsequent FP services visits, a finding supported by the data in this study. Community participants reported that counselling was usually only given at initiation of contraception and then not repeated. Discussing potential side-effects, mechanism of action, adherence information, and drug interactions is essential for accurate and effective use of contraception [59]. If FP clients are not provided with such crucial information unintended pregnancies may occur, which can result in FP clients to assume that the quality of the contraceptive method is inadequate – as participants in this study had mentioned.

HCP explained why counselling and information exchange was so limited. Many HCPs felt constrained by time pressures, patient overload in the clinic, and a lack of training. These factors must be addressed in order to improve the quality of care delivered to FP clients.

The duration spent in a health care facility waiting to receive care is one of the most prominent factors that influences satisfaction with care [73, 74]. Although the contributing factors fall under the structure category, time constraints can be grouped under the process category since it captures the interaction between the provider and client within the health care facility. In this study long waiting times were reported as a significant issue, affecting both access to and the provision of quality FP care, which is supported by the literature in reports about long waiting times for general PHC clients [30]. Specifically related to FP QoC were reports that HCPs have insufficient time available to provide quality care for FP clients. HCP participants especially mentioned that FP care requires more time than other services since extensive counselling and information exchange is required. But with large volumes of clients and the multitasking that is required from nurses, allocating the sufficient time required for quality FP care seems out of reach. This demonstrates how structural components, such as integration, can impact process related factors.

The last element of QoC, follow-up mechanisms, were not mentioned in the data in relation to QoC. This was notable since follow-up mechanisms are considered one of the six elements that form part of the original FP QoC framework [2, 12]. Follow-up mechanisms are crucial to ensure that clients who are engaged in chronic care services, such as family planning, remain in care even if there is a significant period between contact with the health care service – something that has been well demonstrated in the HIV treatment programmes [75, 76]. The follow-up mechanism in the SA public health care sector is based on an appointment booking system where the FP client is issued with a card stating the return date. Yet, earlier studies have shown that many injectable contraceptive users return late for the follow-up appointments, placing them at risk for unintended pregnancy [77]. In a more recent update, it was also found that HCPs keep the appointment card as a measure of protecting those female clients who use contraception covertly from their male partners [49]. Without an effective follow-up mechanism strategy, this component is lacking from the QoC provision in FP service provision.

### Limitations

While the methodology, sampling size, and strategies used were adequate, some limitations should be noted. The perspective employed in this study was from the user and provider experience. Due to the subjective orientation of this study, other larger health system factors that could influence QoC such as financing, procurement, and support services were therefore not explored. Another factor to consider is that this study recruited community participants who may or may not be contraceptive users. Thus, some of the opinions expressed may have been from community members who have not engaged with FP services before, especially the young adolescent group. This can also be considered a strength however, since those community participants who are active contraceptive users were able to freely report their experiences and opinions without fear of repercussion as have been reported in other satisfaction evaluation studies [22].

Qualitative studies are often critiqued for having limited sample sizes and producing non-generalizable findings [78]. The aim of qualitative research is depth and description, and not necessarily breadth and generalisation [79, 80]. The findings presented here should be understood as exploring QoC and its meaning to both users and providers. This approach was useful in identifying key areas where QoC can be improved in this particular setting.

## Conclusion

This study provided some crucial insight into FP QoC in public health facilities in South Africa. These findings reflect how HCPs and community members defined and evaluated QoC. Importantly the findings showed that the current model of integration is having a negative impact on QoC on both the structural and process sides. Careful attention must be paid to how services are being integrated currently and the impact that this integration has on client satisfaction and continued use of FP services. Although FP QoC can be viewed within the broader context of PHC services evaluation, it is important to note that essential QoC aspects specific to FP services were raised in these data. Among these include the fundamental right of clients to choose their own contraceptive method, the need for thorough counselling and information exchange, and the absence of effective follow-up mechanisms. Overall, most of the study participants reported being dissatisfied with the level of FP QoC provided on both the structural and process levels of care. Having identified the major areas of concern that hamper the delivery of quality care presents the opportunity to decrease negative outcomes such as unintended pregnancies and improve positive outcomes such as met need for contraception.

## Data Availability

The datasets generated and analysed during the current study are not publicly available due to the sensitive nature of the data and being qualitative there is a high risk of compromising participant and health care system confidentiality but are available from the corresponding author on reasonable request.

## Abbreviations

FP: family planning
FP QoC: refers to the quality of care provided within family planning service provision.
QoC: Quality of care
SRH: Sexual and reproductive health
HCP: Health care providers
LARC: Long acting-reversible contraceptive
KI: Key informant
FGD: Focus group discussion
NIMART: Nurse Initiated Management of Anti-Retroviral Therapy.
